# A Distinct Glucose Metabolism Signature of Lung Adenocarcinoma With Prognostic Value

**DOI:** 10.3389/fgene.2022.860677

**Published:** 2022-05-09

**Authors:** Ding Li, Jiaming Liang, Wenzhou Zhang, Xuan Wu, Jie Fan

**Affiliations:** ^1^ Department of Pharmacy, The Affiliated Cancer Hospital of Zhengzhou University and Henan Cancer Hospital, Zhengzhou, China; ^2^ Department of Internal Medicine, The Second Affiliated Hospital of Guangzhou Medical University, Guangzhou, China; ^3^ Department of Respiratory and Critical Care Medicine, Zhengzhou University People’s Hospital, Zhengzhou, China; ^4^ Academy of Medical Science, Zhengzhou University, Zhengzhou, China; ^5^ Department of Head Neck and Thyroid Surgery, The Affiliated Cancer Hospital of Zhengzhou University and Henan Cancer Hospital, Zhengzhou, China

**Keywords:** lung adenocarcinoma, glucose metabolism, prognosis, risk signature, biomarker

## Abstract

**Background:** Lung adenocarcinoma (LUAD) remains the most common type of lung cancer and is the main cause of cancer-related death worldwide. Reprogramming of glucose metabolism plays a crucial role in tumorigenesis and progression. However, the regulation of glucose metabolism is still being explored in LUAD. Determining the underlying clinical value of glucose metabolism will contribute in increasing clinical interventions. Our study aimed to conduct a comprehensive analysis of the landscape of glucose metabolism-related genes in LUAD and develop a prognostic risk signature.

**Methods:** We extracted the RNA-seq data and relevant clinical variants from The Cancer Genome Atlas (TCGA) database and identified glucose metabolism-related genes associated with the outcome by correlation analysis. To generate a prognostic signature, least absolute shrinkage and selection operator (LASSO) Cox regression analysis was performed.

**Results:** Finally, ten genes with expression status were identified to generate the risk signature, including FBP2, ADH6, DHDH, PRKCB, INPP5J, ABAT, HK2, GNPNAT1, PLCB3, and ACAT2. Survival analysis indicated that the patients in the high-risk group had a worse survival than those in the low-risk group, which is consistent with the results in validated cohorts. And receiver operating characteristic (ROC) curve analysis further validated the prognostic value and predictive performance of the signature. In addition, the two risk groups had significantly different clinicopathological characteristics and immune cell infiltration status. Notably, the low-risk group is more likely to respond to immunotherapy.

**Conclusion:** Overall, this study systematically explored the prognostic value of glucose metabolism and generated a prognostic risk signature with favorable efficacy and accuracy, which help select candidate patients and explore potential therapeutic approaches targeting the reprogrammed glucose metabolism in LUAD.

## Introduction

As the most common type of lung cancer, lung adenocarcinoma (LUAD) is often diagnosed at an advanced stage with distant metastatic disease (Denisenko et al., 2018). Owing to the substantial advances in the understanding of disease biology, application of predictive biomarkers, refinements in treatment, and therapeutic strategies for LUAD patients ranged from nonselective cytotoxic chemotherapy to personalized precision medicine. Precision medicine is based on validated biomarkers to better classify patients according to probable disease risk, prognosis, and/or treatment response and assists in improving the outcome by combining biomarker measurements and clinical data to a great extent. Therefore, it is important to identify new specific biomarkers to detect more aggressive disease subgroups with poor prognosis (Yuxia et al., 2012). Although a single biomarker has been identified and progressed into the clinic, molecular biomarker panels are still in the discovery stage (Vallée et al., 2014). Biomarker panels consisting of various molecules are promising while genes, proteins, and metabolites work together to promote the development of cancer hallmarks, which could offer a more accurate prediction than a single biomarker (Luo et al., 2018; Song et al., 2021).

Glucose metabolism is reprogrammed in cancer cells to provide energy, biosynthetic precursors, and intermediates for cancer cells (Allen and Locasale, 2018). In addition, reprogrammed glucose metabolism is closely related to the clinical outcome and drug resistance (Boroughs and DeBerardinis, 2015; Faubert et al., 2017). Here, we extracted the RNA-seq profile and relevant clinical information from The Cancer Genome Atlas (TCGA) to systematically and comprehensively analyze the clinical value of glucose metabolism in LUAD. This study aims to provide a distinct signature to better classify patients with different risk scores, as well as potential biomarkers for the use of glucose metabolism and metabolic pathways as therapeutic targets for LUAD.

## Materials and Methods

### Data Collection

The RNA-seq profiles and relevant clinical information were acquired from the University of California, Santa Cruz (UCSC) Xena Browser (https://xenabrowser.net/) on 23 October 2021. The samples with missing clinical information and overall survival (OS) less than 30 days were excluded, and a total of 492 samples were included in the analysis. The other LUAD cohorts, GSE30219, GSE31210, and GSE50081, were downloaded from Gene Expression Omnibus (GEO) (https://www.ncbi.nlm.nih.gov/geo/).

### Estimation of the Glucose Pathways

Fifteen glucose metabolism-related pathways comprising 356 genes were acquired from Molecular Signature Database v7.1 (MSigDB) (http://www.broad.mit.edu/gsea/msigdb/). Single sample gene set enrichment analysis (ssGSEA) from the R package “GSVA” was conducted to determine the activity of each glucose metabolic pathway in LUAD (Yi et al., 2020).

### Generation of a Prognostic Risk Signature

The least absolute shrinkage and selection operator (LASSO) removes coefficients that become zero from the signature by adding a penalty equal to the absolute value of some coefficient magnitudes. Thus, a signature with few coefficients could be created. We randomly split the TCGA LUAD cohort (*n* = 492) into a training (*n* = 368) and testing dataset (*n* = 124) in a ratio of 7–3. A survival analysis for the 356 genes was conducted to select the candidate genes to construct the prognosis signature with *p* < 0.05 based on the log-rank test. Then, LASSO Cox regression analysis was performed with the candidate gene expression profiles from the training dataset to reduce coefficients using the R package “glmnet” (Friedman et al., 2010). Multivariate Cox analysis was followed to identify the most robust markers for the construction of the risk score signature, which included ten genes. The risk score of each sample was calculated as the following formula:

$$\begin{array}{l} Risk\;Score=\;0.293387631789007\mathrm{*GNPNAT}1+0.270363599212865\mathrm{*PLCB}3\\ \;\;\;\;\;\;\;\;\;\;\;\;\;\;\;\;\;\;\;\;\;\;\;\;\;\;\;+0.217376672334296\mathrm{*ACAT}2+\;0.16127906670295\mathrm{*HK}2\\ \;\;\;\;\;\;\;\;\;\;\;\;\;\;\;\;\;\;\;\;\;\;\;\;\;\;\;+0.116014046444865\mathrm{*ADH}6+\left(-0.234392324167846\right)\\ \;\;\;\;\;\;\;\;\;\;\;\;\;\;\;\;\;\;\;\;\;\;\;\;\;\;\;\mathrm{*INPP}5J+\left(-0.202179906028553\right)\mathrm{*PRKCB}\\ \;\;\;\;\;\;\;\;\;\;\;\;\;\;\;\;\;\;\;\;\;\;\;\;\;\;\;+\left(-0.125128962964713\right)\mathrm{*ABAT}+\left(-0.114088018724961\right)\\ \;\;\;\;\;\;\;\;\;\;\;\;\;\;\;\;\;\;\;\;\;\;\;\;\;\;\;\mathrm{*DHDH}+\left(-0.0573408831024442\right)\mathrm{*FBP}2 \end{array}$$

### Prediction of the Immune Response

The response of each sample to anti-PD-1/PD-L1 and anti-CTLA4 immunotherapy was evaluated using the Tumor Immune Dysfunction and Exclusion (TIDE) algorithm according to the gene expression profiles of the LUAD cohort.

### Evaluation of Immune Cell Infiltration

Gene Set Variation Analysis (GSVA), as shown by the R package “GSVA,” carried out a non-parametric unsupervised way to evaluate the underlying pathway activity based on gene expression profiles (Hänzelmann et al., 2013). The marker gene set, consisting of 782 genes that represent 28 immune cell types, was used to assess immune cell infiltration in the tumor microenvironment. The ssGSEA algorithm was performed to estimate the infiltration level of each immune cell type based on the expression profiles (Yoshihara et al., 2013).

### Construction and Evaluation of Nomogram

We constructed a nomogram based on the clinical stage, T stage, and the signature score using the R package “rms.” To assess the application of the nomogram, the R package “ROCsurvival” was performed to construct ROC curves to predict the 1-, 3-, and 5-year OS by the nomogram. The R package “rms” was used to construct calibration curves to assess the accuracy for the prediction of 1-, 3-, and 5-year OS prediction (Li et al., 2021).

### Survival Analysis

The risk score for each sample was used to assess the association between the prognosis of LUAD patients and the risk signature. A Kaplan–Meier curve and log-rank test were performed to compare the differences in OS outcomes between the two risk groups. *p* ＜ 0.05 was set as the significance value. The log-rank test was performed using the R package “survival”, while “surviminer” was performed to plot Kaplan–Meier curves (Zeng et al., 2019).

### Statistical Analysis

Student’s t-tests were performed to determine statistical significance among variables. *p* < 0.05 was defined as statistical significance. All statistical analysis was performed in the R version 4.0.2.

## Results

### Construction of Glucose Metabolism-Related Genes’ Prognostic Signature

**FIGURE 1:**
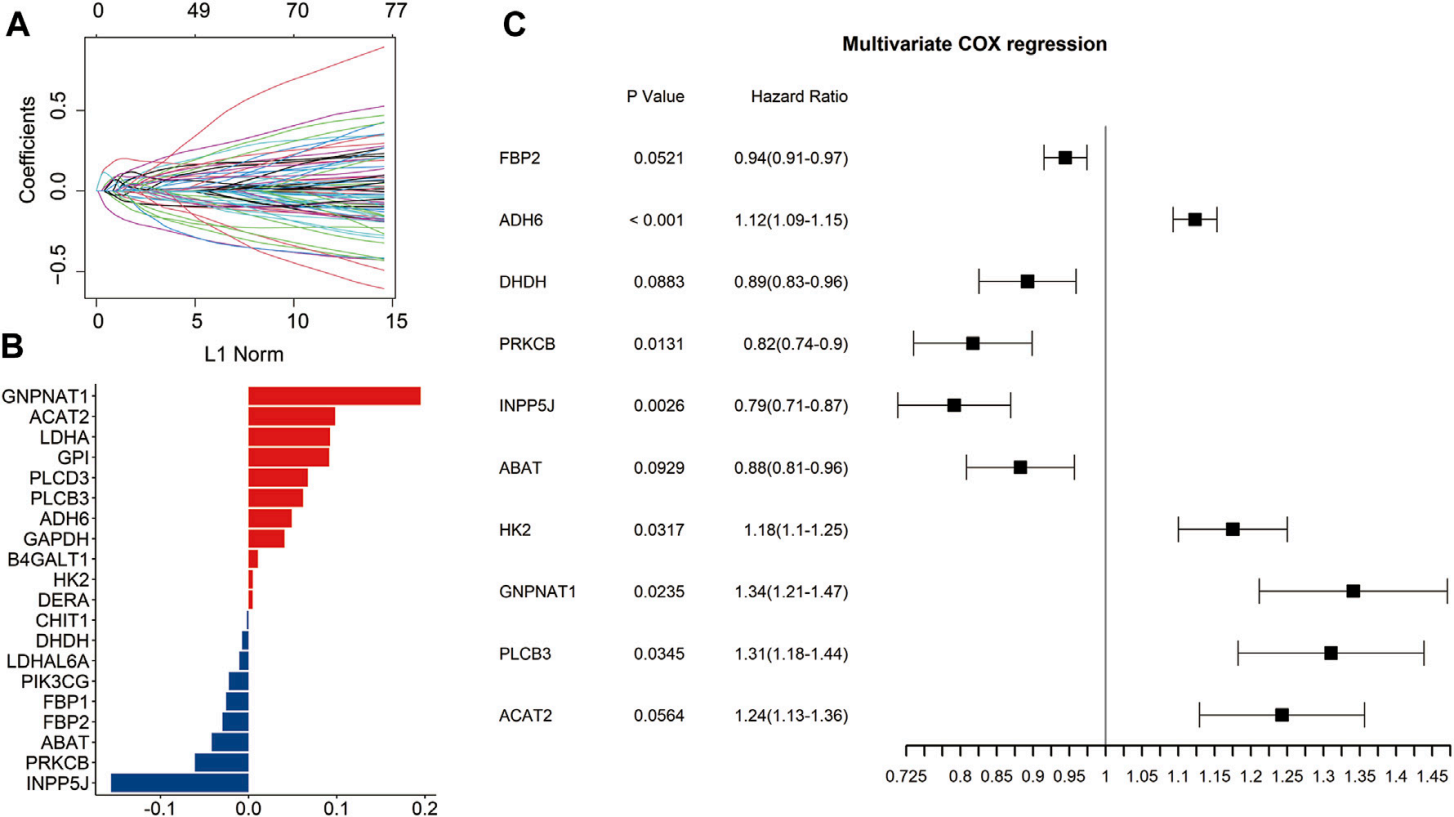
Identification of the prognosis-related genes involved in glucose metabolism. Univariate Cox regression analysis identified 77 genes related to the prognosis of LUAD patients **(A)**. The bar plot showed the coefficients of 20 included glucose metabolism-related genes **(B)**. Multivariate Cox regression analysis identified ten genes to construct the signature **(C)**.

Through univariate Cox regression analysis, 77 genes significantly associated with prognosis were identified from the 356 glucose metabolism-related genes (*p* < 0.05) (Figure 1A). To eliminate collinearity of the variables and avoid over-fitting of the prognostic model, these 77 genes underwent the LASSO regression analysis in the training dataset. Subsequently, 20 candidate genes were identified for further multivariate Cox regression analysis (Figure 1B). Finally, the risk signature was constructed according to the expression levels of ten genes (FBP2, ADH6, DHDH, PRKCB, INPP5J, ABAT, HK2, GNPNAT1, PLCB3, and ACAT2) (Figure 1C).

**FIGURE 2:**
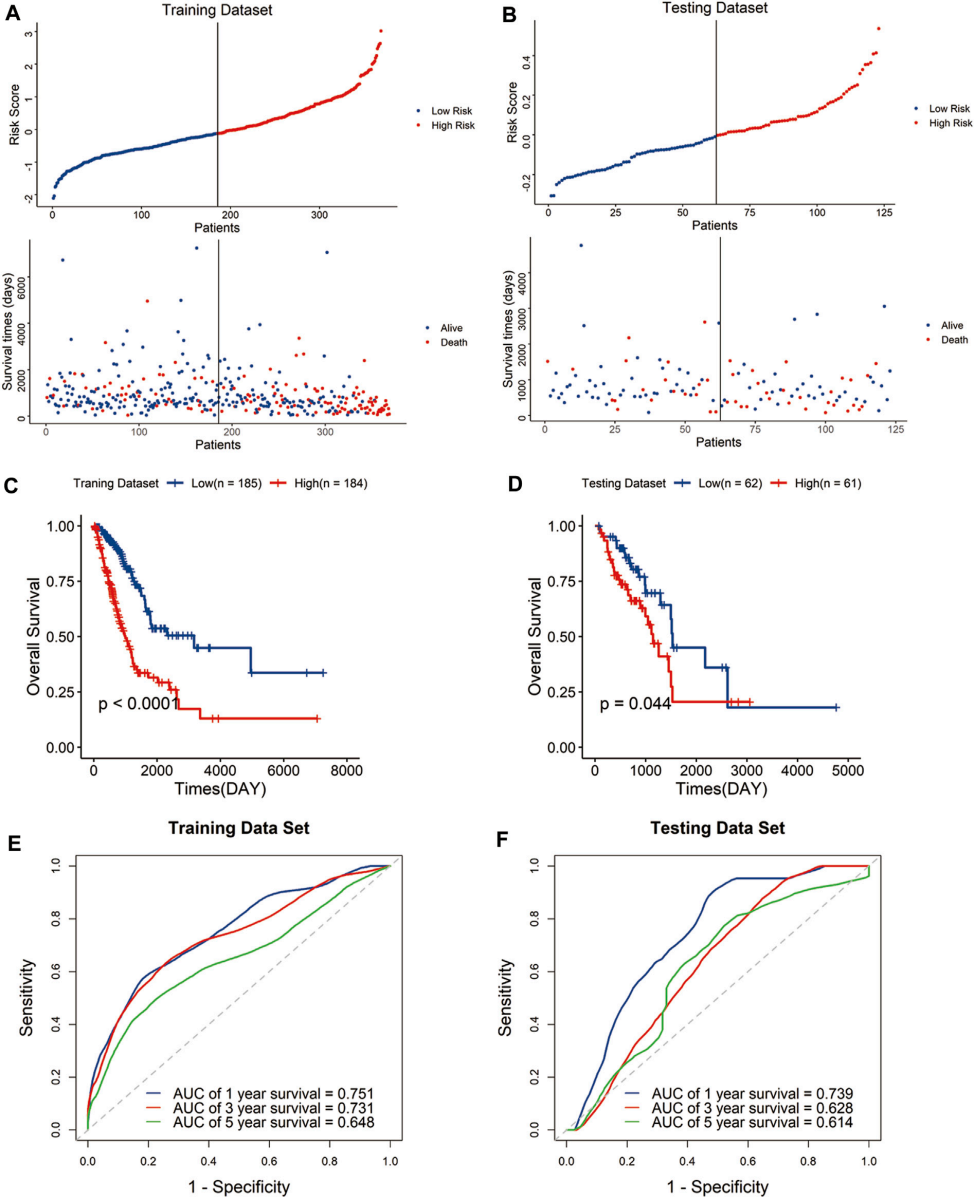
Construction and validation of the risk signature in the TCGA cohort. Distribution of the risk score and survival status in the training dataset **(A)** and testing dataset **(B)**. Kaplan–Meier curves of overall survival for patients with LUAD based on the risk score in the training dataset **(C)** and testing dataset **(D)**. Receiver operating characteristic (ROC) curves of the signature for predicting the 1-, 3-, and 5-year survival in the training dataset **(E)** and testing dataset **(F)**.

The risk score of each sample was calculated with the above formula defined by expression levels of the signature genes and regression coefficients. And, the samples were assigned to high-risk groups and low-risk groups by median risk score both in the training and testing datasets. The scatter plot showed that the high-risk group was associated with a higher mortality rate than the low-risk group (Figures 2A,B).

Kaplan–Meier curves indicated that the high-risk group has significantly poor outcomes compared with the low-risk group (Figures 2C,D). To evaluate the predictive performance of the signature, we performed a time-dependent receiver operating characteristic (ROC) curve based on the risk score. The area under the curves (AUCs) of the 1-, 3-, and 5-year OS were 0.751, 0.731, and 0.648 in the training dataset, and 0.739, 0.628, and 0.614 in the testing dataset, respectively (Figures 2E,F). The results showed the signature displayed great specificity and sensitivity in predicting the prognosis of LUAD patients in TCGA cohort.

### Validation of Glucose Metabolism-Related Genes’ Prognostic Signature Using the GEO Dataset

**FIGURE 3:**
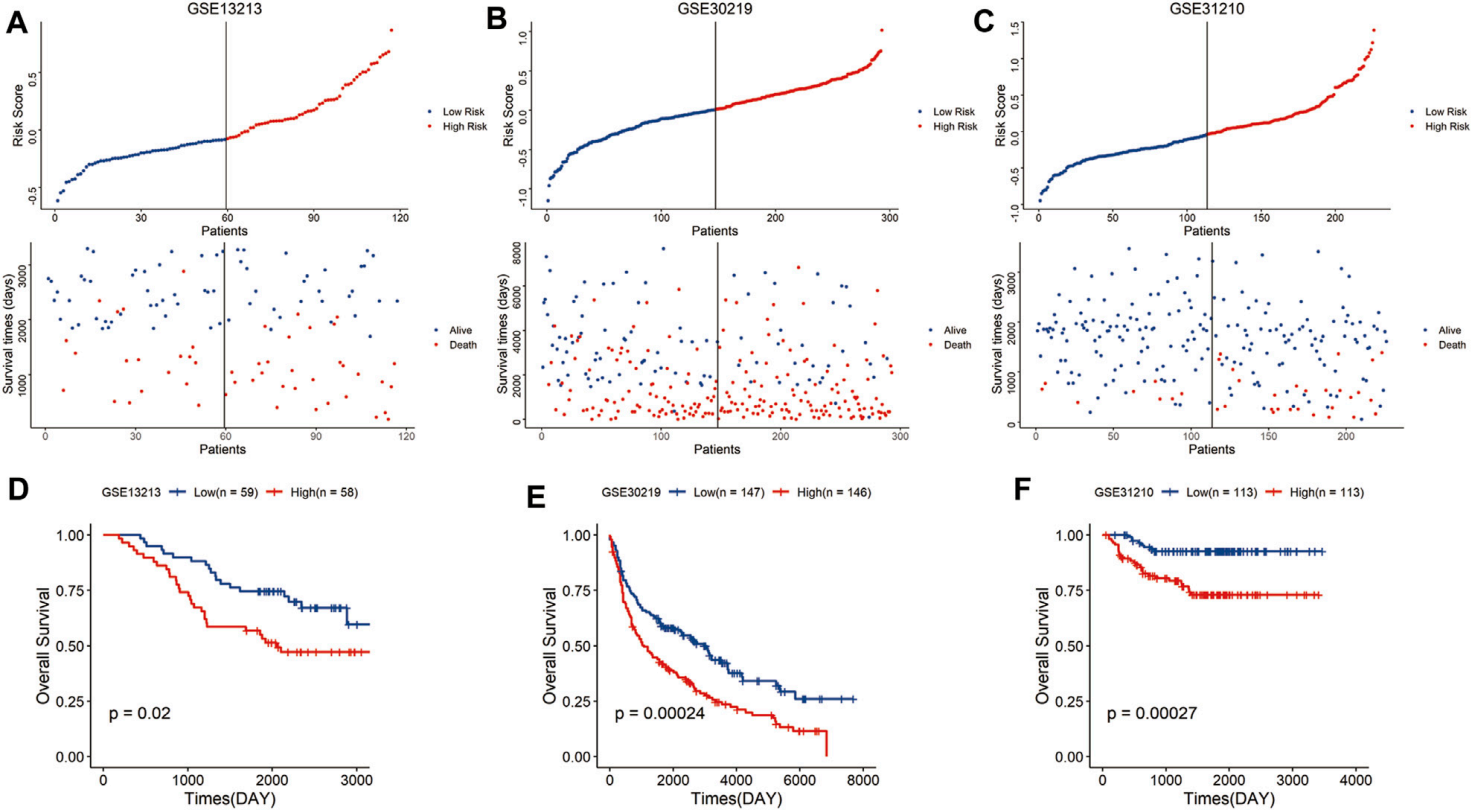
Validation of the risk signature in the GEO cohort. Distribution of the risk score and survival status in the GSE13213 **(A)**, GSE30219 **(B)**, and GSE31210 cohort **(C)**. Kaplan–Meier curves of overall survival for patients with LUAD based on the risk score in the GSE13213 **(D)**, GSE30219 **(E)**, and GSE31210 cohort **(F)**.

To validate the predictive reliability of the signature, we calculated the risk scores of samples in the GEO LUAD cohort using the same formula and similarly classified the samples into high-risk and low-risk groups, and the high-risk group had a higher mortality rate than the low-risk group (Figures 3A–C). As expected, in the GEO database, the high-risk group tended to have a significantly shorter survival time than the low-risk group (Figures 3D–F). Above all, these results showed that the signature had robust and stable predictive power for the LUAD cohort.

### Evaluation of the Signature in Different Subgroups of LUAD Patients

**FIGURE 4:**
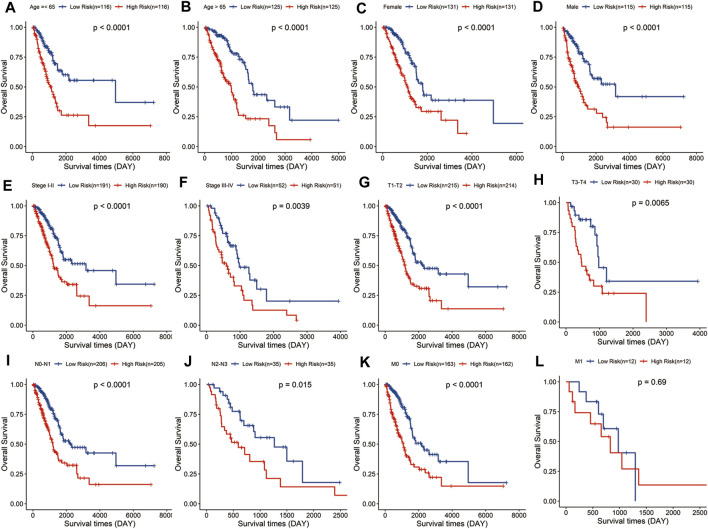
Evaluation of the signature in different subgroups of LUAD patients. Survival analysis in low- and high-risk groups adjusted by clinical variables, including age **(A,B)**, gender **(C,D)**, tumor stage **(E,F)**, and TNM stage **(G–L)**.

Stratified analysis was carried out according to the clinical variables including age (Figures 4A,B), gender (Figures 4C, D), tumor stage (Figures 4E,F), and TNM stage (Figures 4G–L). Kaplan–Meier curve analyses showed that the high-risk group had a worse survival outcome than the low-risk group when stratified by the different clinical features, except for M1, probably because of the small sample size of M1 patients (*n* = 12).

### Construction and Validation of the Nomogram

**FIGURE 5:**
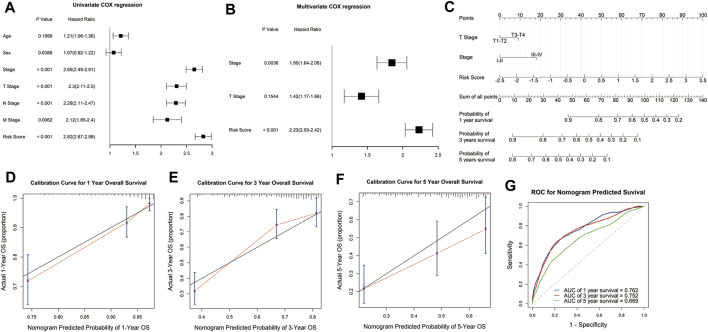
Construction and evaluation of the nomogram. Univariate and multivariate COX regression analysis showed that the risk score was an independent prognostic predictor in the TCGA cohort **(A,B)**. A nomogram was constructed based on the risk score, T stage, and tumor stage **(C)**. Calibration plots of the nomogram for predicting the probability of OS at 1-, 3-, and 5-years in the TCGA cohort **(D–F)**. Time-dependent receiver operating characteristic (ROC) curves for the nomogram to predict 1-, 3-, and 5-year OS in the TCGA dataset **(G)**.

To explore the potential value of the signature in clinical practice, we constructed a nomogram based on the risk score and clinical variables to predict the 1-, 3-, and 5-year survival rates through univariate and multivariate Cox regression analysis. Univariate Cox regression analysis demonstrated that risk score, tumor stage, and TNM stage were significantly associated with the survival of LUAD patients (Figure 5A). Multivariate Cox regression analysis showed that the risk score was an independent prognostic factor for LUAD patients after adjusting for these clinical parameters, although tumor and T stage were also independent (Figure 5B). Then we constructed the nomogram with the risk score, tumor, and T stage for their independent prognostic ability and clinical accessibility (Figure 5C). Calibration plots revealed that the nomogram showed perfect concordance between the observed and predicted survival rates at 1-, 3-, and 5-years (Figures 5D–F). The time-dependent ROC curves demonstrated that the nomogram had excellent predictive accuracy in predicting the 1-, 3-, and 5-year survival of LUAD patients. The AUCs for 1-, 3-, and 5-year survival was 0.762, 0.752, and 0.669, which indicated that the nomogram has robust and stable ability to predict the survival of LUAD patients (Figure 5G).

### Correlation Between Immune Cell Infiltration and Risk Score

**FIGURE 6:**
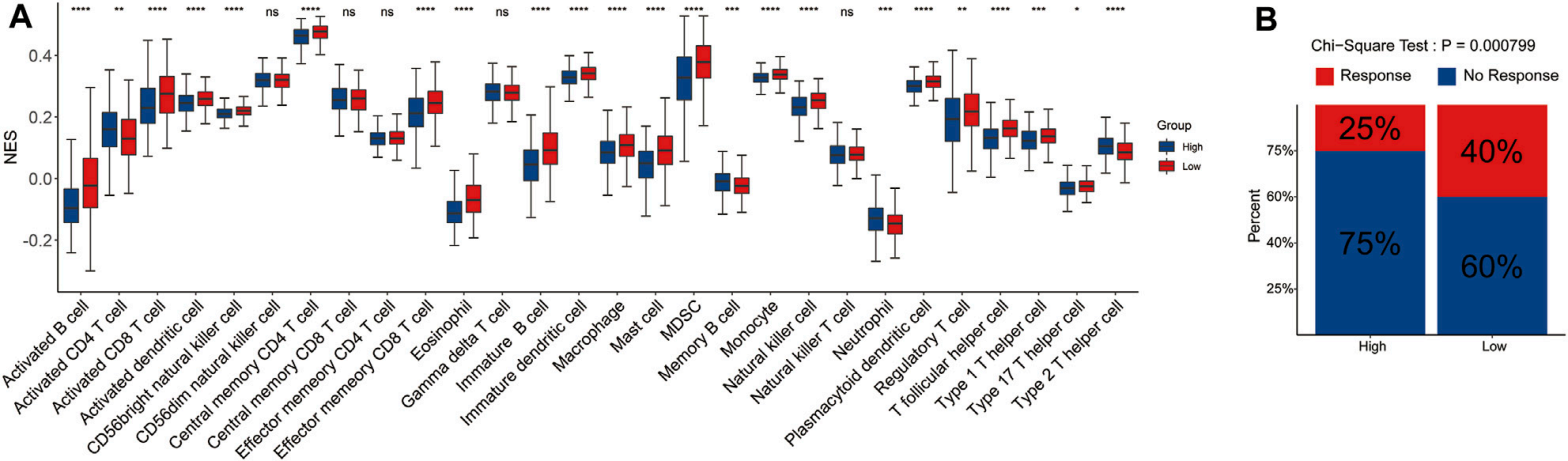
Correlation between tumor-infiltrating immune cell and risk score. The infiltrating level of each of the 28 tumor-infiltrating immune cells between the high- and low-risk groups **(A)**. The rate of response to immunotherapy between the two risk groups **(B)**.

To explore the potential correlation of the signature with the immune microenvironment, we performed the CIBERSORT algorithm to evaluate the infiltrating level of immune cells in the tumor microenvironment and made comprehensive comparisons with the risk score. The results showed that the proportions of 28 immune cell types were significantly different between the two risk groups, and the low-risk group tended to have significantly higher infiltrating levels of the most immune cell types than the high-risk group, which may represent an intrinsic feature that can characterize individual differences (Figure 6A).

Furthermore, we also evaluated the difference in the response rate of immunotherapy between the two risk groups. Samples in the low-risk group had a higher response rate to immunotherapy than those in the high-risk group (Figure 6B). The aforementioned results indicated that signature could predict the immune cell infiltration level and the response to immunotherapy in LUAD.

### Gene Set Enrichment Analysis

**FIGURE 7:**
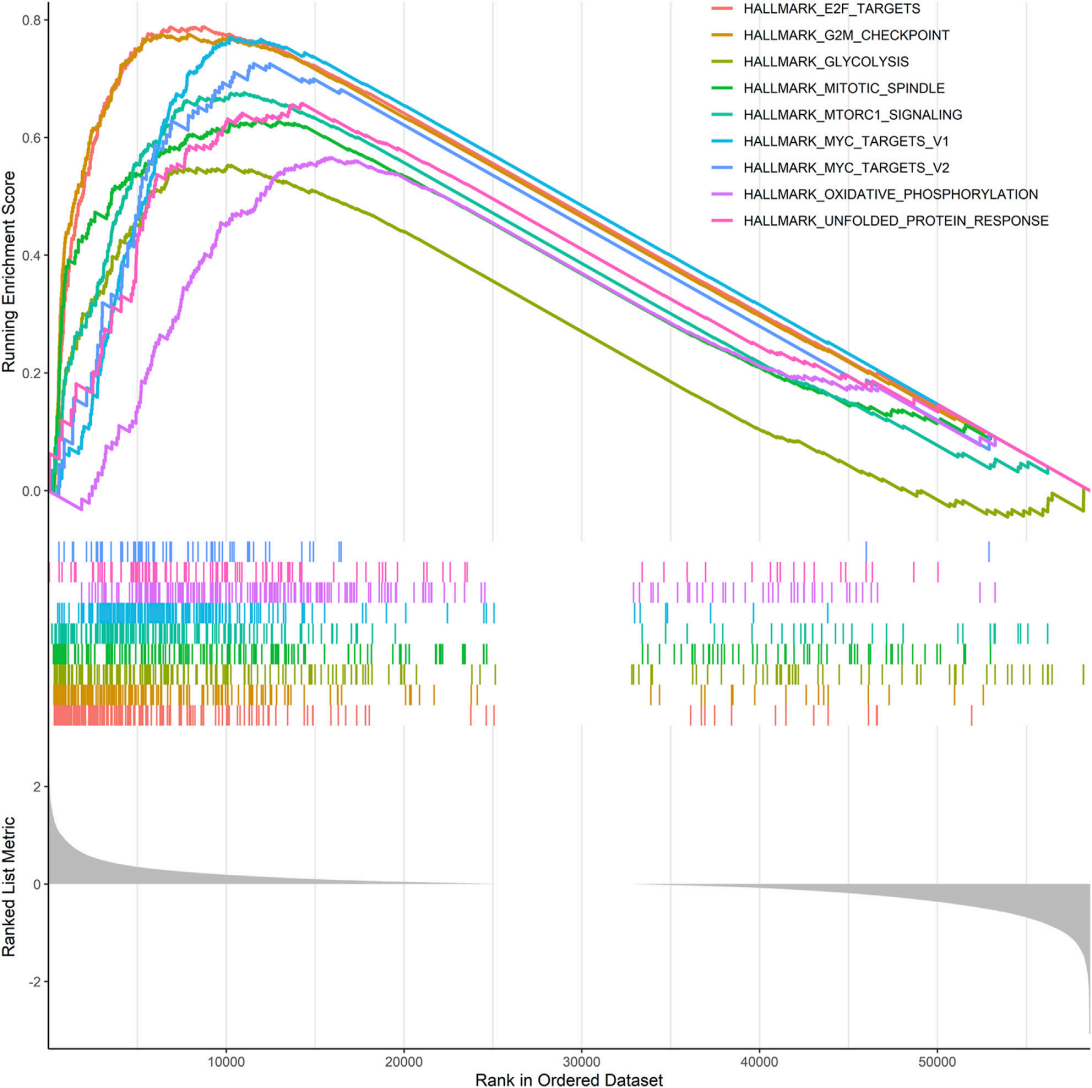
GSEA was performed using the HALLMARK collection.

Given that risk scores were inversely associated with prognosis in patients with LUAD, further functional annotation was performed between the two risk groups using GSEA. The result showed that enriched gene sets of the HALLMARK collection in the high-risk group were mainly involved in tumor-related pathways, including E2F, G2/M checkpoint, glycolysis, mTORC1, MYC, oxidative phosphorylation, and unfolded protein response, which are closely related to the malignant proliferation of tumor cells (Figure 7).

## Discussion

LUAD is the most common histological subtype of NSCLC, often with the presence of specific genetic mutations for further molecular stratification (Xiong et al., 2020). Since the patients at an early stage could have a favorable prognosis, most patients have developed distant metastasis at the first time of diagnosis, with poor survival (Denisenko et al., 2018). Risk stratification is important to assess the prognosis of patients, which may promote the development of new strategies for LUAD management. Furthermore, prognostic prediction plays an important role in treatment selection and the identification of potential prognostic biomarkers (Wang et al., 2020; Yi et al., 2021).

Tumorigenesis and progression are primely required for metabolic reprogramming in cancer cells (Taubes 2012). Cancer cells could alter their fluxes via various metabolic pathways to meet increased biosynthetic and bioenergetic demands and alleviate oxidative stress required for cancer cell proliferation and survival (Boroughs and DeBerardinis, 2015). In recent years, there has been a growing interest in developing cancer genetic analysis for patient stratification in combination with therapies that target metabolism (Hay 2016). Although it is well known that metabolic reprogramming is a hallmark of cancer, regulation of glucose metabolism in LUAD is still being explored, and identifying the underlying clinical value of glucose metabolism in LUAD phenotype may contribute to increased clinical interventions. In addition, there is an urgent need to identify new strategies for patient stratification with easier access to gene abnormality detection in cancers, which will promote the efficiency and velocity of translation from basic research to clinical practice (Wettersten et al., 2017; Qin et al., 2020). However, studies regarding transcriptome-wide analysis on the correlation between glucose metabolism and LUAD are limited. We evaluated the correlation between glucose metabolism-related pathways and clinical characteristics as well as the immune phenotype in LUAD. The ssGSEA was conducted to calculate the enrichment score of each gene set regulating glucose metabolism-related pathways, and the results showed that the citrate cycle (TCA cycle) pathway had the highest score, whereas the enrichment score of the ascorbate and aldarate metabolism pathways are the lowest (Supplementary Figure S1). To better understand the clinical significance of the glucose metabolism-related pathways in LUAD, we compared the discrepancies in the pathways between different subgroups of LUAD. The result showed that the samples with the N2-N3 stage had a significantly higher enrichment score in glyoxylate and dicarboxylate metabolism than that of the ones with the N0-N1 stage (*p* ＜ 0.05), whereas there is no significant difference in the pathways in the subgroups stratified by T and M stage (Supplementary Figures S2–S4). In addition, the citrate cycle (TCA cycle), glyoxylate and dicarboxylate, and pentose phosphate metabolism pathways have a significantly elevated enrichment score in tumor stage III-IV LUAD samples compared with tumor stage I-II LUAD samples (*p* ＜ 0.05) (Supplementary Figure S5). The results demonstrated that the specific glucose metabolism pathway was significantly associated with the specific subgroup of LUAD patients.

Here, we first introduce a glucose metabolism-related prognosis signature for the malignancy of LUAD and the survival of LUAD patients. From 356 glucose metabolism-related genes involved in 15 pathways, we finally included ten genes, of which their expressions were significantly associated with prognosis, to construct a risk signature. The prognostic risk signature showed great predictive ability both in the training and testing datasets and was an independent indicator for the prognosis of LUAD patients.

Furthermore, we also evaluated the distribution trends of glucose metabolism-related pathways between the two risk groups in the TCGA database (Supplementary Figure S6). It can be seen that among the 15 pathways, the ascorbate and aldarate metabolism pathway, citrate cycle (TCA cycle) pathway, fructose and mannose metabolism pathway, galactose metabolism pathway, glyoxylate and dicarboxylate metabolism pathway, pentose and glucuronate interconversions pathway, pentose phosphate pathway, and starch and sucrose metabolism pathway increased with an increase in the risk score, suggesting that these pathways’ imbalances had a significantly positive correlation with tumor development. The results may provide some insight in the glucose metabolism scape of tumor development.

The most included genes in the risk signature have been reported to play important roles in tumorigenesis and progression in various cancer types, which enhance the predictive performance of the signature.

Among the ten genes, fructose-1,6-bisphosphatase 2 (FBP2) has been demonstrated to inhibit glycolysis and growth in gastric cancer cells (Li et al., 2013). Alcohol dehydrogenase (ADH) had shown potential prognostic values in pancreatic adenocarcinoma and hepatocellular carcinoma (Liao et al., 2017; Liu et al., 2020). DHDH had been reported to be included in a metabolism-related prognostic signature for hepatocellular carcinoma (Yang et al., 2021). PRKCB has also been reported to be included in the prognostic signature for adult T-cell leukemia/lymphoma and prostate cancer (Kataoka et al., 2018; Daniunaite et al., 2021). INPP5J regulates AKT1-dependent breast cancer growth and metastasis and predicts recurrence in lung adenocarcinoma (Ooms et al., 2015; Zhang et al., 2020). ABAT and HK2 have been reported to play crucial roles in cancer metabolism, progression, and therapeutic resistance of cancers (Jansen et al., 2015; Garcia et al., 2019; Shen et al., 2020). GNPNAT1 and PLCB3 had shown the independent prognostic potential in NSCLC (Zhang et al., 2019; Zheng et al., 2020). ACAT2 could promote cell proliferation and associated with malignant progression in colorectal cancer (Weng et al., 2020). The evidence mentioned earlier demonstrated that these included signature genes might play vital roles in cancer, and their roles in LUAD should be further explored.

Previous studies have demonstrated that immune cell infiltration and immune checkpoints are correlated with the response rate of immunotherapy in LUAD (Bodor et al., 2020). We assessed the correlations between the risk signature and immune cell infiltration. The proportions of 28 immune cell types in the tumor microenvironment were significantly different between the two risk groups, and the low-risk group tended to have significantly higher infiltrating levels of the most immune cell types than the high-risk group. Notably, the glucose metabolism-related signature was significantly correlated to CD4^+^ and CD8^+^ T cells, and the samples in the high-risk group tended to have a lower number of CD8^+^ T cells and a higher number of CD4^+^ T cells. The result indicated that patients of higher risk tend to have an unfavorable tumor-infiltrating lymphocyte pattern. Moreover, the signature was also significantly associated with innate immune cell types, including macrophages, monocytes, and NK cells, which is consistent with the results of previous research that showed tryptophan metabolic adaptation in lung cancer was related to evasion of innate immune by cancer cells (Cassetta and Pollard, 2018; Dejima et al., 2021). Since immune checkpoint inhibitors have shown promising anti-tumor effects by reversing the immunosuppressive effects of tumors, the expression of immune checkpoints has attracted widespread attention as a biomarker for identifying patients with LUAD to receive immunotherapy. Immune checkpoints could be used to predict the efficacy of immune checkpoint blockade and have been proven to be a biomarker for identifying patients who can benefit from immunotherapy in several cancer types. In this study, we analyzed the association between the signature genes and immune checkpoints. The expression of the ten signature genes was significantly associated with the expression of the four checkpoint markers, PD-1, PD-L1, PD-L2, and CTLA-4 (Supplementary Figure S7). The findings showed that the risk signature based on glucose metabolism-related genes was involved in the altered immune microenvironment of LUAD.

Overall, we constructed a risk signature based on the glucose metabolism-related genes for the prognosis, malignancy, and immune phenotype of LUAD, which might provide a better understanding of the glucose metabolic role in immune phenotype and carcinogenesis. This study also suggested that glucose metabolism could be a potential target and that the glycolytic inhibitor combined with immunotherapy maybe a novel strategy for LUAD treatment.

## Data Availability

The data that support the findings of this study are available from the corresponding author upon reasonable request.

## Supplementary Material

Supplementary Figure 1 Enrichment score of each gene set regulating glucose metabolism-related pathway.

Supplementary Figure 2 Correlation between the enrichment score of each pathway and the LUAD patients with N stage.

Supplementary Figure 3 Correlation between the enrichment score of each pathway and the LUAD patients with T stage.

Supplementary Figure 4 Correlation between the enrichment score of each pathway and the LUAD patients with M stage.

Supplementary Figure 5 Correlation between the enrichment score of each pathway and the LUAD patients with tumor stage.

Supplementary Figure 6 Correlation between the enrichment score of each pathway and the risk score.

Supplementary Figure 7 Correlation between the expression of checkpoints and each gene of the signature.

## References

[B1] Allen A. E. Locasale J. W. (2018). Glucose Metabolism in Cancer: The Saga of Pyruvate Kinase Continues. Cancer Cell 33, 337–339. 10.1016/j.ccell.2018.02.008 29533776 PMC6237085

[B2] Bodor J. N. Boumber Y. Borghaei H. (2020). Biomarkers for Immune Checkpoint Inhibition in Non-Small Cell Lung Cancer (NSCLC). Cancer 126, 260–270. 10.1002/cncr.32468 31691957 PMC7372560

[B3] Boroughs L. K. DeBerardinis R. J. (2015). Metabolic Pathways Promoting Cancer Cell Survival and Growth. Nat. Cel. Biol. 17, 351–359. 10.1038/ncb3124

[B4] Cassetta L. Pollard J. W. (2018). Targeting Macrophages: Therapeutic Approaches in Cancer. Nat. Rev. Drug Discov. 17, 887–904. 10.1038/nrd.2018.169 30361552

[B5] Daniunaite K. Bakavicius A. Zukauskaite K. Rauluseviciute I. Lazutka J. R. Ulys A. (2021). Promoter Methylation of PRKCB, ADAMTS12, and NAALAD2 Is Specific to Prostate Cancer and Predicts Biochemical Disease Recurrence. Int. J. Mol. Sci. 22, 6091. 10.3390/ijms22116091 34198725 PMC8201120

[B6] Dejima H. Hu X. Chen R. Zhang J. Fujimoto J. Parra E. R. (2021). Immune Evolution from Preneoplasia to Invasive Lung Adenocarcinomas and Underlying Molecular Features. Nat. Commun. 12, 2722. 10.1038/s41467-021-22890-x 33976164 PMC8113327

[B7] Denisenko T. V. Budkevich I. N. Zhivotovsky B. (2018). Cell Death-Based Treatment of Lung Adenocarcinoma. Cell Death Dis. 9, 117. 10.1038/s41419-017-0063-y 29371589 PMC5833343

[B8] Faubert B. Li K. Y. Cai L. Hensley C. T. Kim J. Zacharias L. G. (2017). Lactate Metabolism in Human Lung Tumors. Cell 171, 358–371. e359. 10.1016/j.cell.2017.09.019 28985563 PMC5684706

[B9] Friedman J. Hastie T. Tibshirani R. (2010). Regularization Paths for Generalized Linear Models via Coordinate Descent. J. Stat. Softw. 33, 1–22. 10.18637/jss.v033.i01 20808728 PMC2929880

[B10] Garcia S. N. Guedes R. C. Marques M. M. (2019). Unlocking the Potential of HK2 in Cancer Metabolism and Therapeutics. Curr. Med. Chem. 26, 7285–7322. 10.2174/0929867326666181213092652 30543165

[B11] Hänzelmann S. Castelo R. Guinney J. (2013). GSVA: Gene Set Variation Analysis for Microarray and RNA-Seq Data. BMC Bioinformatics 14, 7. 10.1186/1471-2105-14-7 23323831 PMC3618321

[B12] Hay N. (2016). Reprogramming Glucose Metabolism in Cancer: Can it Be Exploited for Cancer Therapy? Nat. Rev. Cancer 16, 635–649. 10.1038/nrc.2016.77 27634447 PMC5516800

[B13] Jansen M. P. H. M. Sas L. Sieuwerts A. M. Van Cauwenberghe C. Ramirez-Ardila D. Look M. (2015). Decreased Expression of ABAT and STC2 Hallmarks ER-Positive Inflammatory Breast Cancer and Endocrine Therapy Resistance in Advanced Disease. Mol. Oncol. Jun 9, 1218–1233. 10.1016/j.molonc.2015.02.006

[B14] Kataoka K. Iwanaga M. Yasunaga J.-i. Nagata Y. Kitanaka A. Kameda T. (2018). Prognostic Relevance of Integrated Genetic Profiling in Adult T-Cell Leukemia/lymphoma. Blood 131, 215–225. 10.1182/blood-2017-01-761874 29084771 PMC5757690

[B15] Li D. Liang J. Cheng C. Guo W. Li S. Song W. (2021). Identification of m6A-Related lncRNAs Associated with Prognoses and Immune Responses in Acute Myeloid Leukemia. Front. Cel. Dev. Biol. 9, 770451. 10.3389/fcell.2021.770451

[B16] Li H. Wang J. Xu H. Xing R. Pan Y. Li W. (2013). Decreased Fructose-1,6-Bisphosphatase-2 Expression Promotes Glycolysis and Growth in Gastric Cancer Cells. Mol. Cancer 12, 110. 10.1186/1476-4598-12-110 24063558 PMC3849177

[B17] Liao X. Huang R. Liu X. Han C. Yu L. Wang S. (2017). Distinct Prognostic Values of Alcohol Dehydrogenase mRNA Expression in Pancreatic Adenocarcinoma. Ott 10, 3719–3732. 10.2147/ott.s140221

[B18] Liu X. Li T. Kong D. You H. Kong F. Tang R. (2020). Prognostic Implications of Alcohol Dehydrogenases in Hepatocellular Carcinoma. BMC Cancer 20, 1204. 10.1186/s12885-020-07689-1 33287761 PMC7720489

[B20] Luo P. Yin P. Hua R. Tan Y. Li Z. Qiu G. (2018). A Large‐Scale, Multicenter Serum Metabolite Biomarker Identification Study for the Early Detection of Hepatocellular Carcinoma. Hepatology 67, 662–675. 10.1002/hep.29561 28960374 PMC6680350

[B21] Ooms L. M. Binge L. C. Davies E. M. Rahman P. Conway J. R. W. Gurung R. (2015). The Inositol Polyphosphate 5-Phosphatase PIPP Regulates AKT1-Dependent Breast Cancer Growth and Metastasis. Cancer Cell 28, 155–169. 10.1016/j.ccell.2015.07.003 26267533

[B22] Qin C. Yang G. Yang J. Ren B. Wang H. Chen G. (2020). Metabolism of Pancreatic Cancer: Paving the Way to Better Anticancer Strategies. Mol. Cancer 19, 50. 10.1186/s12943-020-01169-7 32122374 PMC7053123

[B23] Shen C. Xuan B. Yan T. Ma Y. Xu P. Tian X. (2020). m6A-Dependent Glycolysis Enhances Colorectal Cancer Progression. Mol. Cancer 19, 72. 10.1186/s12943-020-01190-w 32245489 PMC7118901

[B24] Song J. Ma S. Sokoll L. J. Eguez R. V. Höti N. Zhang H. (2021). A Panel of Selected Serum Protein Biomarkers for the Detection of Aggressive Prostate Cancer. Theranostics 11, 6214–6224. 10.7150/thno.55676 33995654 PMC8120218

[B25] Taubes G. (2012). Ravenous for Glucose. Science 335, 31. 10.1126/science.335.6064.31 22223789

[B26] Vallée A. Le Loupp A.-G. Denis M. G. (2014). Efficiency of the Therascreen RGQ PCR Kit for the Detection of EGFR Mutations in Non-Small Cell Lung Carcinomas. Clinica Chim. Acta 429, 8–11. 10.1016/j.cca.2013.11.014

[B27] Wang Q. Li M. Yang M. Yang Y. Song F. Zhang W. (2020). Analysis of Immune-Related Signatures of Lung Adenocarcinoma Identified Two Distinct Subtypes: Implications for Immune Checkpoint Blockade Therapy. Aging (Albany NY) 12, 3312–3339. 10.18632/aging.102814 32091408 PMC7066911

[B28] Weng M. Zhang H. Hou W. Sun Z. Zhong J. Miao C. (2020). ACAT2 Promotes Cell Proliferation and Associates with Malignant Progression in Colorectal Cancer. Ott 13, 3477–3488. 10.2147/ott.s238973

[B29] Wettersten H. I. Aboud O. A. Lara P. N. Jr. Weiss R. H. (2017). Metabolic Reprogramming in Clear Cell Renal Cell Carcinoma. Nat. Rev. Nephrol. 13, 410–419. 10.1038/nrneph.2017.59 28480903

[B30] Xiong Y. Lei J. Zhao J. Feng Y. Qiao T. Zhou Y. (2020). Gene Expression-Based Clinical Predictions in Lung Adenocarcinoma. Aging (Albany NY) 12, 15492–15503. 10.18632/aging.103721 32756002 PMC7467359

[B31] Yang X. Liu Q. Zou J. Li Y.-k. Xie X. (2021). Identification of a Prognostic Index Based on a Metabolic-Genomic Landscape Analysis of Hepatocellular Carcinoma (HCC). Cmar 13, 5683–5698. 10.2147/cmar.s316588

[B32] Yi M. Nissley D. V. McCormick F. Stephens R. M. (2020). ssGSEA Score-Based Ras Dependency Indexes Derived from Gene Expression Data Reveal Potential Ras Addiction Mechanisms with Possible Clinical Implications. Sci. Rep. 10, 10258. 10.1038/s41598-020-66986-8 32581224 PMC7314760

[B33] Yi M. Li A. Zhou L. Chu Q. Luo S. Wu K. (2021). Immune Signature-Based Risk Stratification and Prediction of Immune Checkpoint Inhibitor's Efficacy for Lung Adenocarcinoma. Cancer Immunol. Immunother. 70, 1705–1719. 10.1007/s00262-020-02817-z 33386920 PMC8139885

[B34] Yoshihara K. Shahmoradgoli M. Martínez E. Vegesna R. Kim H. Torres-Garcia W. (2013). Inferring Tumour Purity and Stromal and Immune Cell Admixture from Expression Data. Nat. Commun. 4, 2612. 10.1038/ncomms3612 24113773 PMC3826632

[B35] Yuxia M. Zhennan T. Wei Z. (2012). Circulating miR-125b Is a Novel Biomarker for Screening Non-Small-Cell Lung Cancer and Predicts Poor Prognosis. J. Cancer Res. Clin. Oncol. 138, 2045–2050. 10.1007/s00432-012-1285-0 22806310 PMC11824208

[B36] Zeng L. Fan X. Wang X. Deng H. Zhang K. Zhang X. (2019). Bioinformatics Analysis Based on Multiple Databases Identifies Hub Genes Associated with Hepatocellular Carcinoma. Cg 20, 349–361. 10.2174/1389202920666191011092410

[B37] Zhang T. Song X. Liao X. Wang X. Zhu G. Yang C. (2019). Distinct Prognostic Values of Phospholipase C Beta Family Members for Non-Small Cell Lung Carcinoma. Biomed. Res. Int. 2019, 4256524. 10.1155/2019/4256524 31080817 PMC6475572

[B38] Zhang Y. Fan Q. Guo Y. Zhu K. (2020). Eight-Gene Signature Predicts Recurrence in Lung Adenocarcinoma. Cbm 28, 447–457. 10.3233/cbm-190329

[B39] Zheng X. Li Y. Ma C. Zhang J. Zhang Y. Fu Z. (2020). Independent Prognostic Potential of GNPNAT1 in Lung Adenocarcinoma. Biomed. Res. Int. 2020, 8851437. 10.1155/2020/8851437 33178836 PMC7648248

