# Supplementary figures and images for: A Distinct Glucose Metabolism Signature of Lung Adenocarcinoma With Prognostic Value
Source: Front Genet. 2022 May 9;13:860677. doi: 10.3389/fgene.2022.860677 (PMC9125243; doi:10.3389/fgene.2022.860677)

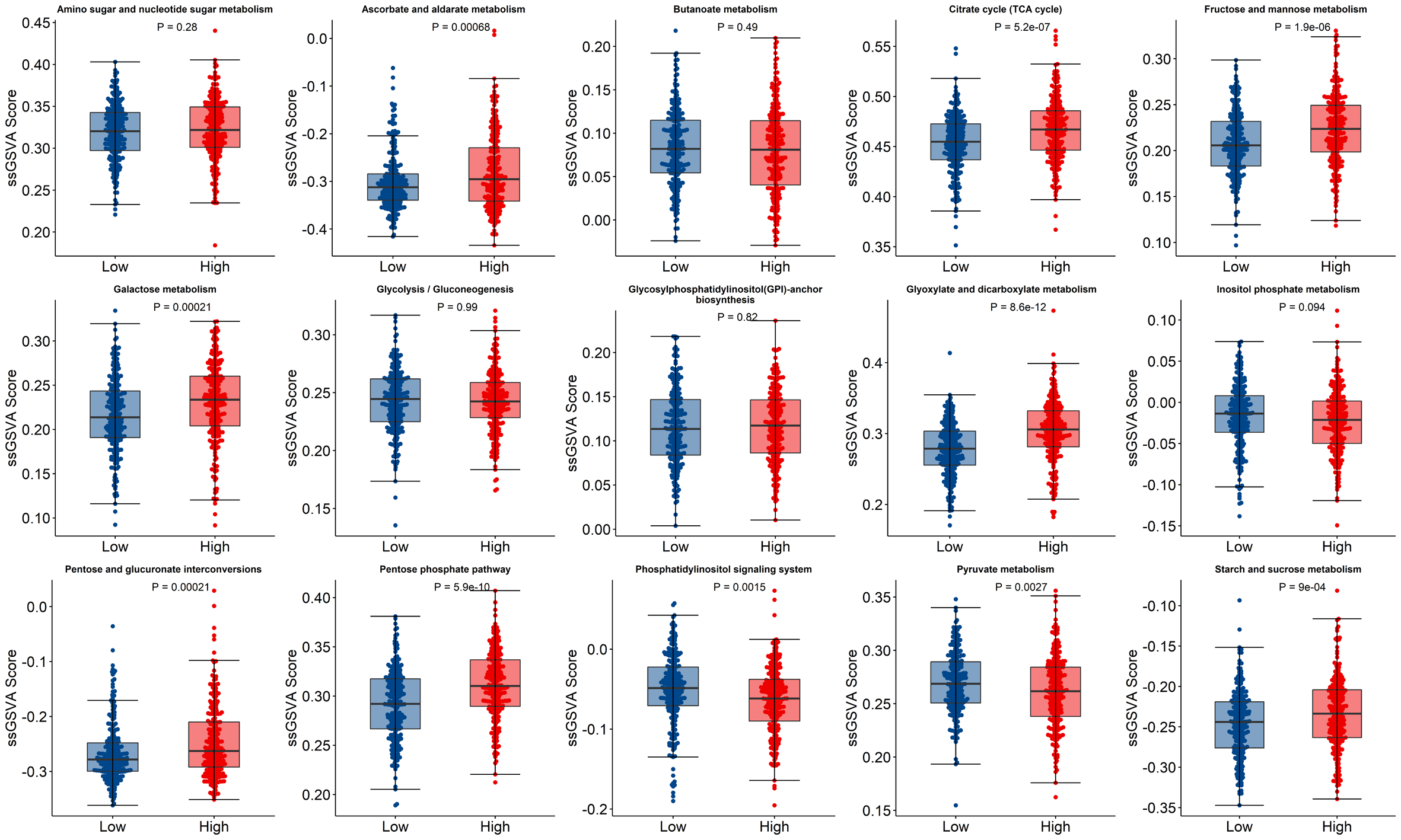

Supplement: Supplementary file 1 [file Image6.TIF]

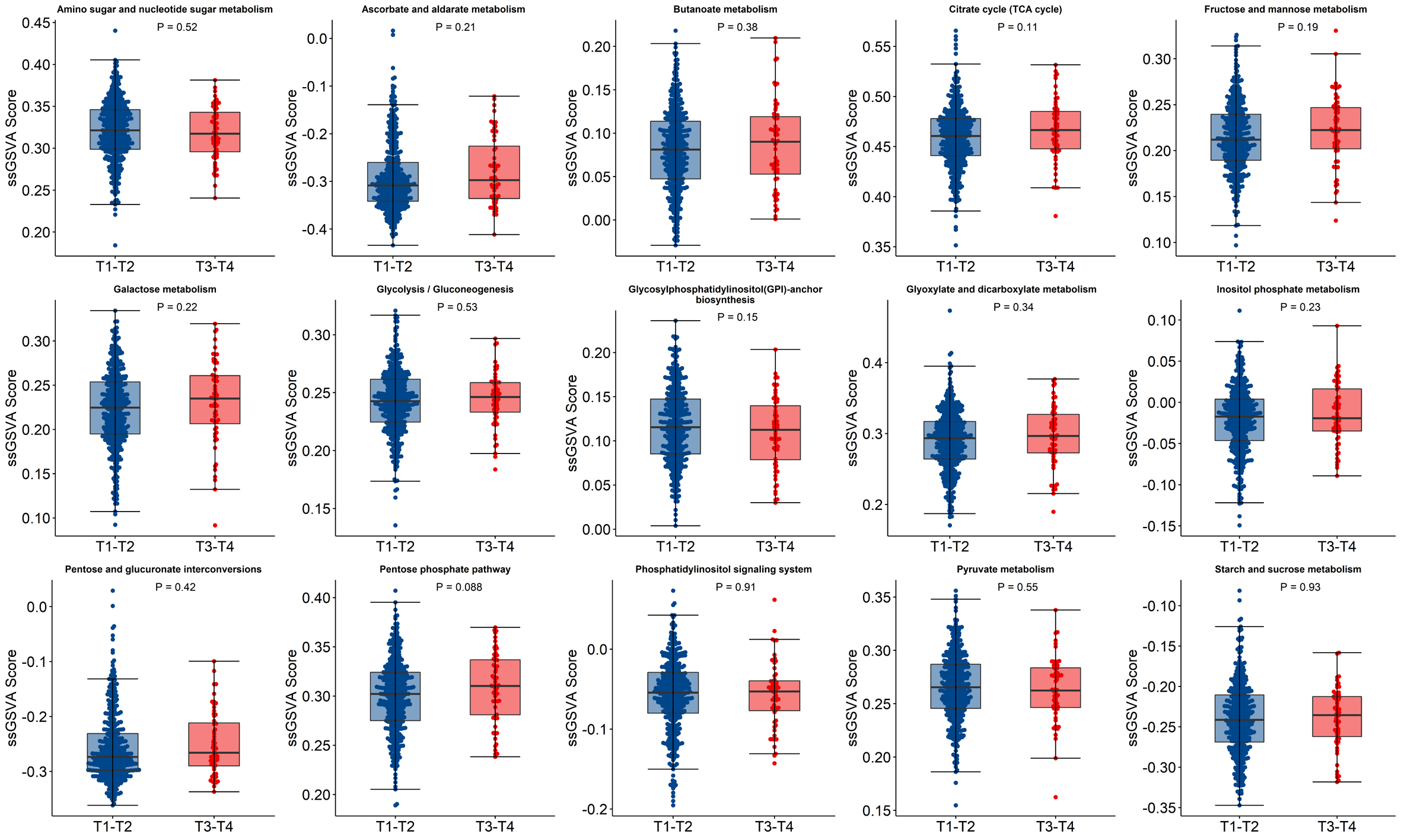

Supplement: Supplementary file 2 [file Image3.TIF]

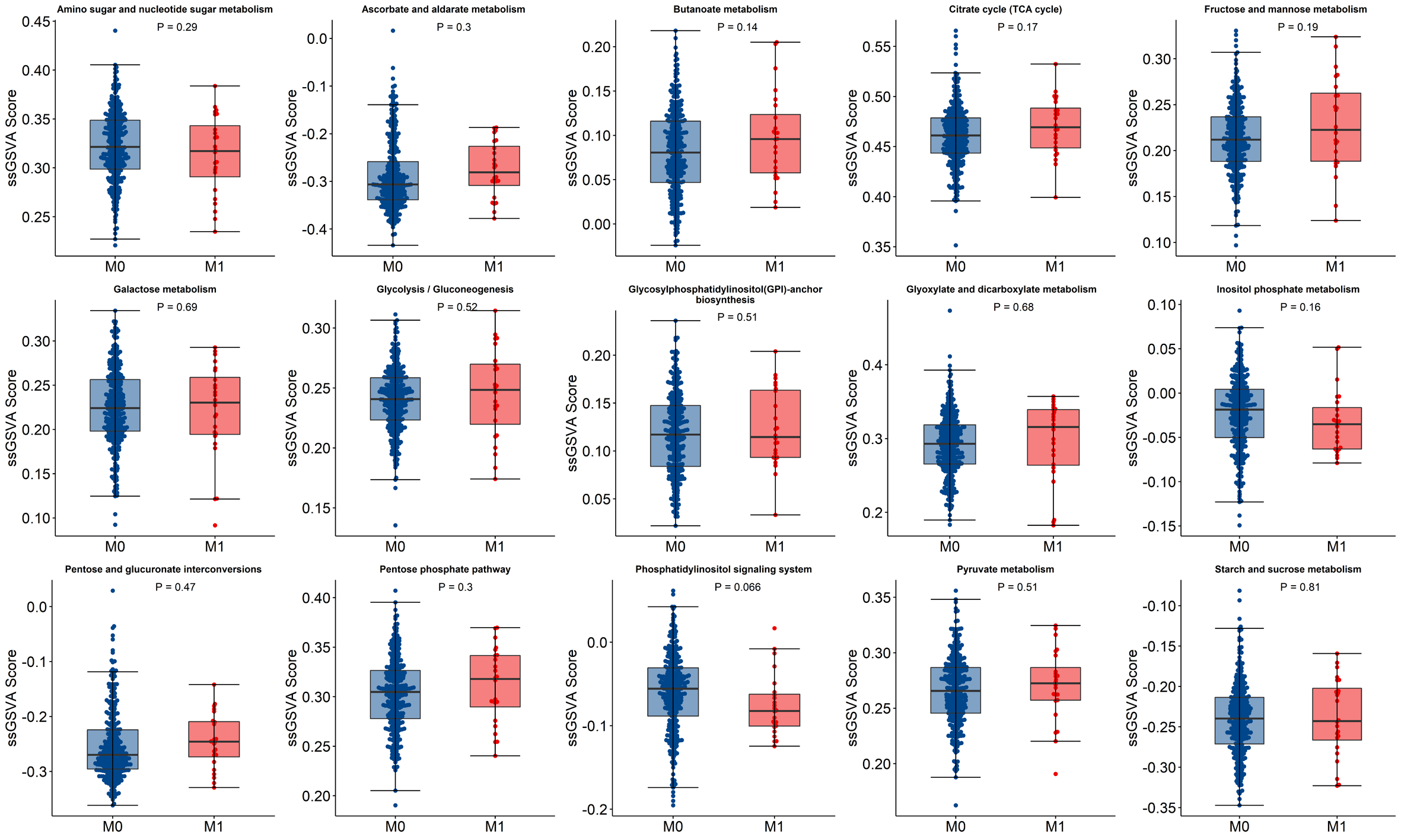

Supplement: Supplementary file 3 [file Image4.TIF]

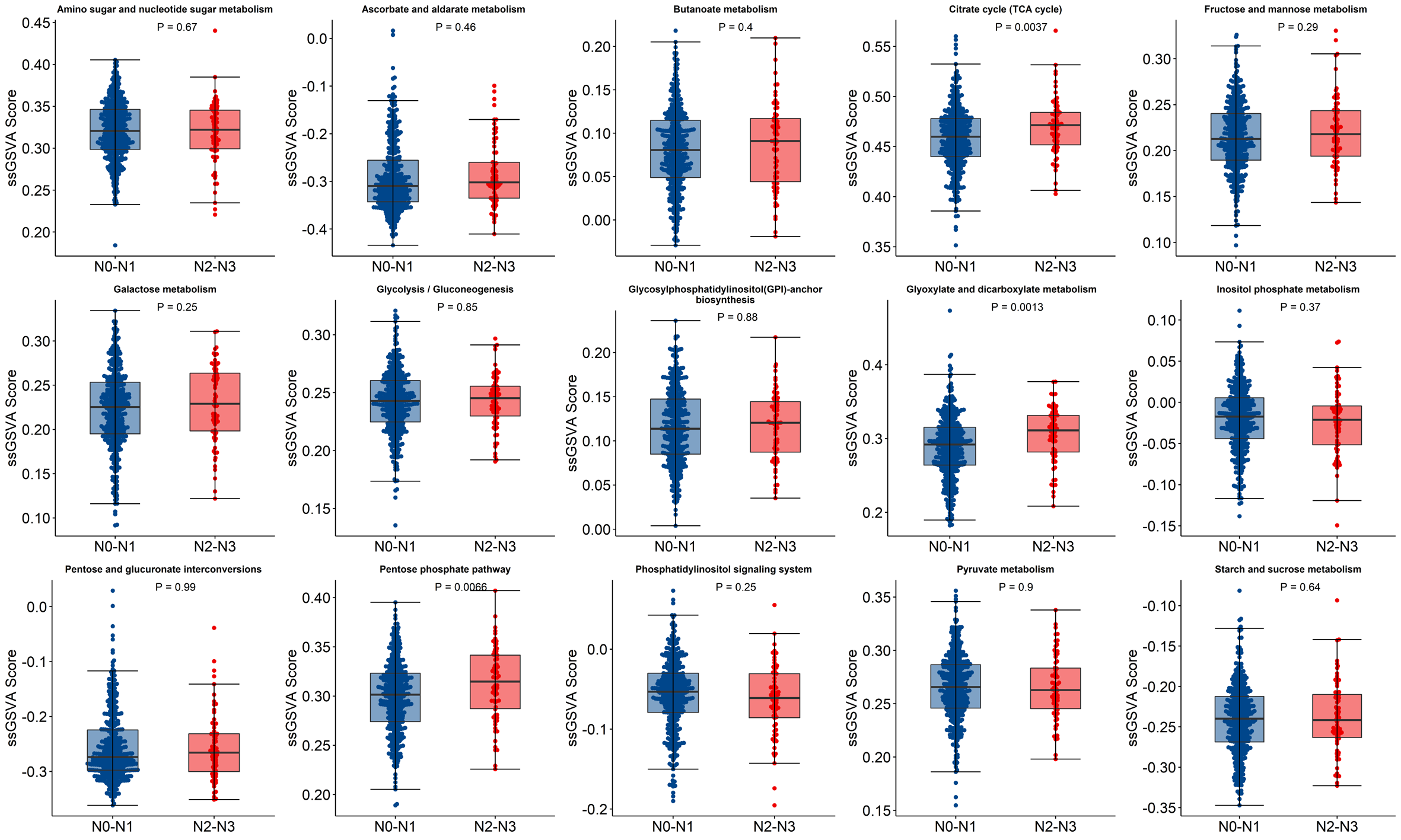

Supplement: Supplementary file 4 [file Image2.TIF]

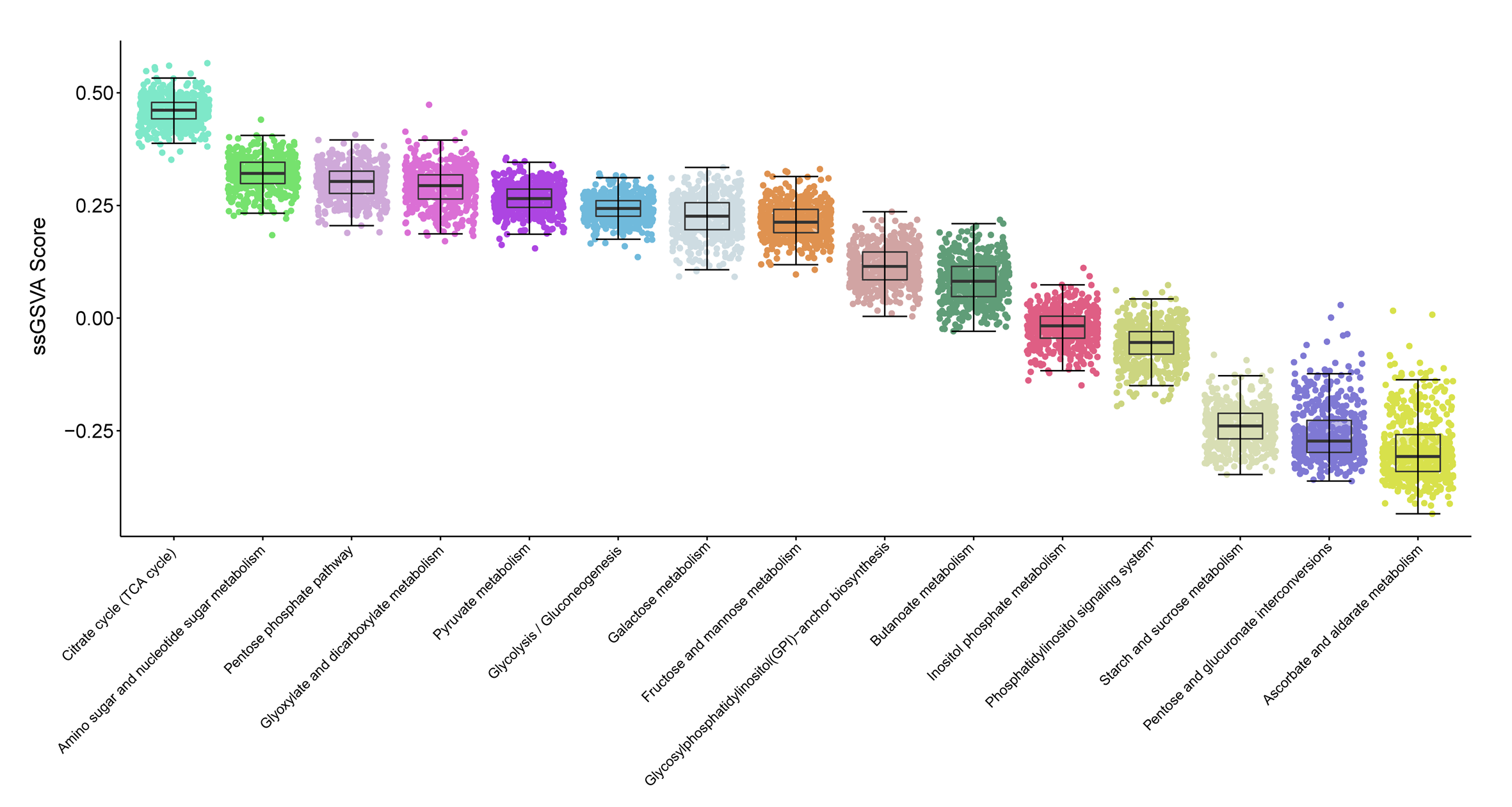

Supplement: Supplementary file 5 [file Image1.TIF]

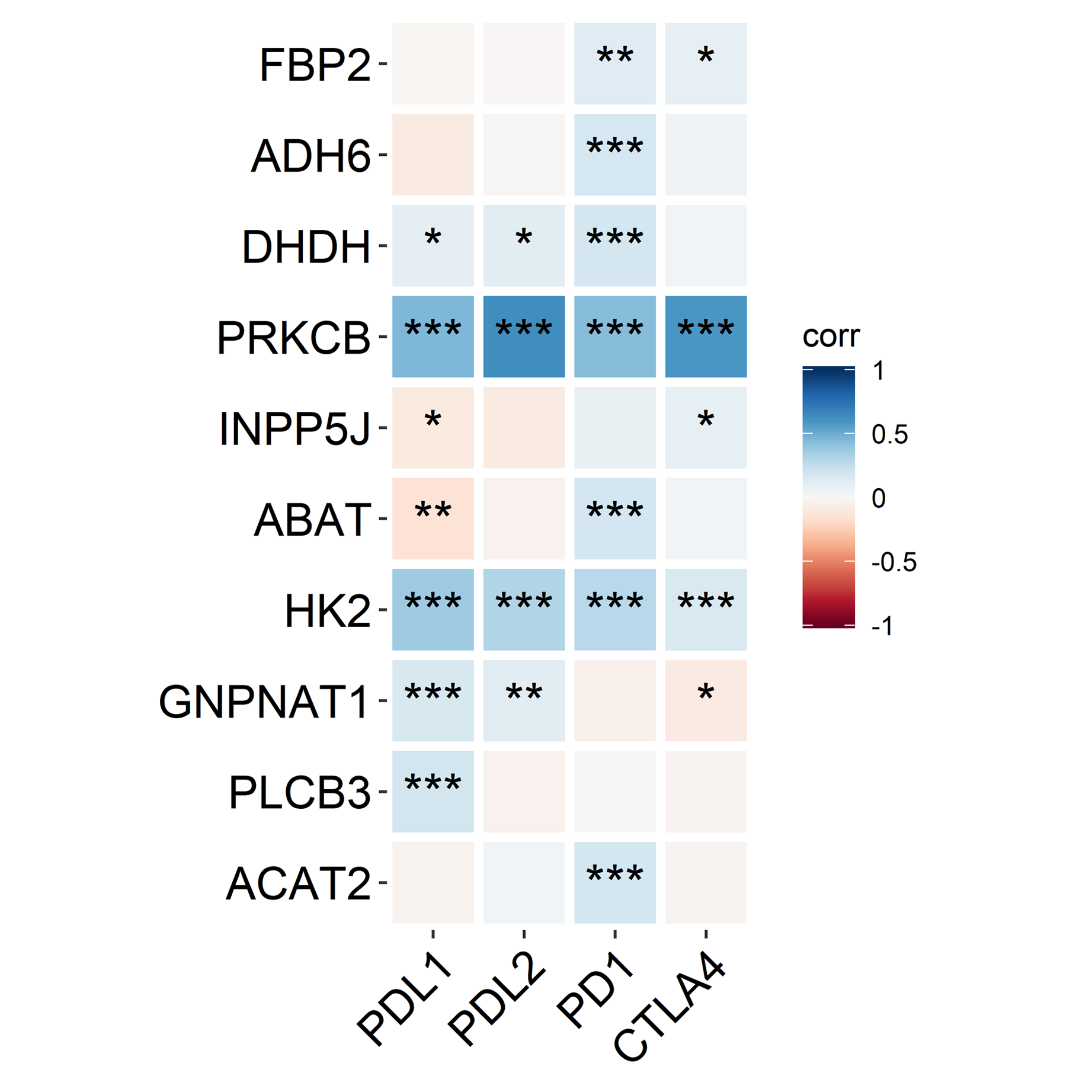

Supplement: Supplementary file 6 [file Image7.TIF]

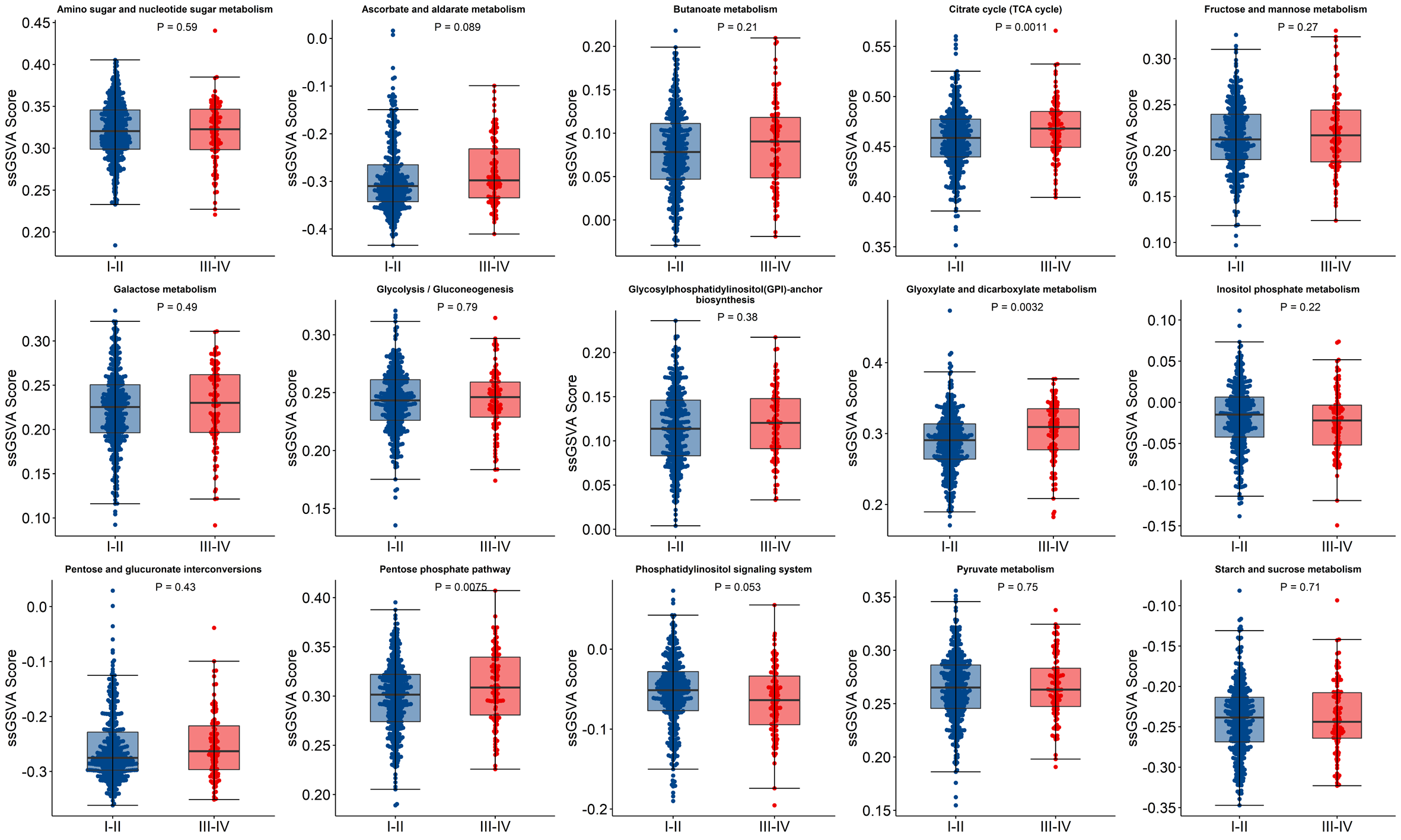

Supplement: Supplementary file 7 [file Image5.TIF]
